# Self-rated health of Latino day laborers during the COVID-19 pandemic: a cross-sectional study

**DOI:** 10.21203/rs.3.rs-4810665/v1

**Published:** 2024-08-29

**Authors:** Jan Catindig, John Atkinson, Ana Llamas, Maria Eugenia Fernandez-Esquer

**Affiliations:** The University of Texas Health Science Center at Houston; The University of Texas Health Science Center at Houston; The University of Texas Health Science Center at Houston; The University of Texas Health Science Center at Houston

**Keywords:** self-rated health, trust, Latino, COVID-19 pandemic, mental health, networks

## Abstract

**Background:**

This study assesses the relationship between trust in sources of information in regard to the Coronavirus Disease 2019 (COVID-19) pandemic and self-rated health (SRH) in a sample of Latino Day Laborers (LDLs) and explores whether these associations were mediated by mental health measures.

**Methods:**

A rapid needs assessment survey was conducted with 300 LDLs, recruited at randomly selected hiring locations in Houston, Texas, during November and December 2021. Two measures of trust were developed, and SRH was measured by a single item. Depression, anxiety, and stress were measured by previously validated scales. We applied the product of coefficients approach to assess our mediation model. Using Hayes’ PROCESS version 4.2 in SPSS, linear regression models were generated simultaneously to assess the total effect of trust on SRH, effect of trust on the mental health mediator, and effect of the mediator on SRH, controlling for trust. Demographic characteristics were entered as covariates.

**Results:**

Greater trust in formal sources of information (such as Spanish-speaking television networks) as well as greater levels of anxiety and depression were associated with lower SRH. There were no significant associations between formal trust and mental health measures. As such, the association between formal trust and decreased SRH was not mediated by mental health. Trust in informal sources of information (conversations with friends, family, and coworkers) was not significantly associated with SRH or mental health. Higher levels of depression and anxiety, however, were associated with lower SRH.

**Conclusions:**

LDLs’ perception of their health was inversely associated with their level of trust in formal sources of information and with greater levels of depression and anxiety. Nevertheless, these pathways were independent of each other. The results indicate the direct impact of COVID-19 public information on subjective well-being, a relationship that merits further exploration.

## Background

The COVID-19 pandemic has influenced general subjective well-being, particularly among individuals negatively affected by the accompanying economic downturn. In the United States (US), individuals who report poor health are more likely to be members of low-income, ethnic minority populations [1]. Contributing to this health disparity are environmental, social, and health-related exposures, social determinants, and cultural-related stressors [1], which may have an independent as well as a synergistic influence on health. For instance, individuals who experienced housing insecurity during the COVID-19 pandemic were shown to have increased psychological distress and decreased self-rated health (SRH) [2].

Latinos are considered a population vulnerable to COVID-19 due to structural and environmental determinants, social conditions, and personal risk factors that have an impact on their well-being [3]. According to the Centers for Disease Control and Prevention COVID-19 demographics and data on trends, 1,182,801 deaths occurred between January 1, 2020, and April 30, 2024, and the percentage of deaths among Hispanics was 14.71% (174,019 individuals) [4].

Although there is variation in Latino vulnerability to COVID-19, some groups, such as Latino Day Laborers (LDLs), have been disproportionately affected by the pandemic. LDLs are a subgroup of mainly recently immigrated Latino men who engage in unregulated and informal work and are especially vulnerable to environmental stressors and occupational hazards due to the harsh, unfavorable conditions they confront at work. Due to these circumstances, Latino immigrant workers, including LDLs, had increased exposure to COVID-19 and were more susceptible to its economic and health-related repercussions than were other ethnic groups [5].

The high COVID-19 infection and mortality rates experienced by LDLs also are partly due to barriers they encounter in seeking healthcare. As an example, Latinos across the US are targeted by policy rulings that may have an impact on their willingness to use medical care [6]. In the context of the COVID-19 pandemic, LDLs have experienced limited access to preventive health care and federal programs [3]. In relation to preventive health care, mistrust has been reported to be one of the key barriers to COVID-19 diagnostic testing among all Latinos, including LDLs [5,7]. Contributing to mistrust is the inadequate, unreliable, and inconsistent COVID-19 information that has been circulated to and among marginalized communities, which has influenced their seeking healthcare services [7] and could have had an impact on their overall health. The underutilization of mental health services among Latinos may be influenced by facets of their culture [8]. In addition, misinformation from various social sources, including family members, social networks, and Spanish-language media and news channels, such as Telemundo and Univision, have been a barrier to COVID-19 vaccine knowledge and uptake among Latino communities [9].

### Self-Rated Health

SRH is a reliable and valid measure of an individual’s general health [10,11,12,13]. Assessment of SRH involves an individual’s perception of health in the psychological, social, and physical realms [1] and the subjective assessment of various factors, including physical functioning and social engagement [14]. SRH is commonly measured as a single item that asks an individual to rate his or her general health, e.g., “In general, would you say your health is excellent, very good, good, fair, or poor?” [11,14].

SRH has been used extensively in public health-related, sociological, and physical health-related research studies [12,13,14] and found to be predictive of mortality, morbidity, individuals’ health behaviors, and health status [12,13,14,15]. SRH is also associated with mental health, as individuals who report decreased SRH have increased odds of also reporting depression [16]. Studies have reported a strong correlation between poor SRH and premature mortality and the risk of developing chronic health outcomes, relative to an individual who reports excellent SRH [1,17]. SRH is an independent predictor of survival, particularly for individuals who report having very good or excellent SRH [14].

### Trust

Trust in people and institutions is a measure of social capital [18] and degree of connectedness [19] that may have an impact on an individual’s perceived health and may ultimately affect health outcomes. According to the American Psychological Association, trust is an individual’s confidence and reliance on a person’s or a group’s dependability and is a vital aspect of interpersonal relationships [20*]. Institutional* trust is individuals’ belief that governmental institutions will implement appropriate policies that are aligned with the expected behaviors of individuals and entities [21]. *Interpersonal* trust is individuals’ expectations that other individuals will engage in mutual enhancement of well-being without causing any harm [21]. By extension, *informational* trust may be understood as confidence in the dependability and credibility of institutional and interpersonal information sources.

Trust, measured as trust in people, feeling of reciprocity, and feeling safe in the neighborhood, is a dimension of social capital and has been positively associated with good SRH and psychological well-being, which indicates that individuals with higher levels of trust reported better health outcomes compared to individuals with lower levels [22]. Measuring trust is critical to understanding the health of Latinos, specifically LDLs, as LDLs tend to report lower levels of trusted social relationships; one study found that 36% of LDLs reported a lack of trusted friends, and 33% reported a lack of trusted coworkers [10]. This limited connectedness is likely to influence Latino SRH and overall health.

Trust in health information and health advice [23] was critical during the COVID-19 pandemic. This type of trust includes formal and informal health information sources, such as traditional media, local and national organizations, social media, and interpersonal communication [24,25,26]. Lack of trust in the accuracy and reliability of COVID-19 diagnostic testing results contributed to underserved Latino communities’ reluctance to participate in COVID-19 mitigation practices, such as vaccination [7]. In addition, mistrust in the health system was a barrier to healthcare utilization, and misinformation regarding COVID-19 was associated with an increased risk of exposure to COVID-19 among both documented and undocumented Mexican immigrants [27]. Undocumented Latino immigrants’ lack of documentation and identification was a significant barrier to their COVID-19 testing, and distrust in the handling of their personal data was a concern in their participation in COVID-19 diagnostic testing [7]. Distrust in public institutions, in general, complicates efforts to assist individuals who are experiencing distress due to disasters [28], including the COVID-19 pandemic.

In addition, individuals’ attitudes and knowledge concerning the COVID-19 pandemic were influenced by broadcast television news [29]. Latinos in the US depend on news broadcast on television channels, with an estimated 89% of Latino immigrants able to access Spanish news sources to some extent, and these sources were often preferred by Latinos as a means to obtain information relating to the COVID-19 pandemic [29]. Latinos had higher levels of trust in the information disseminated in Spanish by broadcasters who were of similar ethnicity, and Spanish television news sources had an average viewer count of 712,827 for Telemundo and 1,104,233 for Univision [29]. A comparison of news disseminated by television networks shows that the COVID-19 pandemic had been covered more thoroughly by Cable News Network (CNN), as it reported more preventive response strategies, such as social distancing and potential lockdowns, than Fox News network [30]. One study found that individuals who trusted formal sources of information demonstrated lower preventive behaviors and higher engagement in risky behaviors, possibly due to the network’s political stance [31]. Additionally, in the US, individuals who had greater trust in television sources had lower engagement in protective behaviors [26], and increased viewing of traditional media was associated with lower COVID-19 vaccination uptake [32]. Another study found that mental distress (i.e., feeling nervous, worried, depressed, and loss of interest) was not significantly associated with trust in television sources concerning COVID-19 information [33].

Moreover, social media has been shown to be one of the main sources of information regarding the COVID-19 pandemic. False health information regarding COVID-19 proliferated on social media and created turmoil and panic among individual consumers [34]. Exposure to COVID-19 news and information on social media has been reported to be associated with adverse mental health outcomes and poor SRH, with individuals who were exposed to frequent use of social media as reporting higher levels of anxiety and depression [16] in the context of the COVID-19 pandemic. Further, the more that individuals trusted COVID-19 information from social media sources, the higher their levels of anxiety as compared to individuals who had less trust in social media, as many social media sources provided inaccurate and unreliable information regarding the COVID-19 pandemic [34]. Moreover, individuals who trusted social media for COVID-19 information reported higher odds of severe mental distress compared to those who did not trust social media [33].

### Mental Health

In the context of the COVID-19 pandemic, social, mental, and physical health outcomes, including anxiety, depression, emotional distress, and obesity, increased among adults in the US [35,36]. Moreover, due to the lack of clear and sufficient COVID-19 information, poorer mental health among immigrants in the US has intensified and worsened [37]. Research provides evidence of the association between mental health, SRH, and trust in the context of the COVID-19 pandemic. For instance, one study explored trust in institutions (e.g., healthcare system, news media) and found that older adults who had a higher score on trust were also more likely to report a higher score on perceived health [38].

Latino immigrants have been susceptible to poor mental health due to the disproportionate economic burden they have confronted, including financial setbacks, such as loss of employment and housing insecurity, and reported experiencing mental health problems during the COVID-19 pandemic, including symptoms consistent with anxiety disorders and depression [39]. In addition, Latino immigrants reported that fear of contracting the COVID-19 virus and social isolation contributed to their mental health concerns [39]. Undocumented Latino immigrants were at an increased risk for the adverse health outcomes of COVID-19, including psychological distress due to the nature of their job as essential workers, salary reductions, and workplace shutdowns, resulting in financial insecurity [40]. In addition to these economic burdens, misinformation regarding COVID-19 resulted in overall mistrust, and individuals demonstrated having anxiety and stress, manifested by a fear of going out, and these mental health symptoms led to undesirable health behaviors [41].

### Demographic Factors in the Context of the COVID-19 Pandemic

Even when Latinos were disproportionately affected by the COVID-19 pandemic, not all members of this group had the same fate. Variations are related to demographic characteristics and certain factors, as discussed below. In our review of the literature, we identified a gap in what is known about the influence of demographic factors specific to LDLs, as previous studies focused primarily on broader Latino populations and other immigrant communities.

### Correlates of Trust

Establishing trust between community members and testing administrators was a crucial facilitator in COVID-19 diagnostic testing among Latinos in the US [7]. In a study on the influence of trust on compliance with COVID-19 contact tracers among various racial and ethnic groups, Latinos exhibited the least amount of trust, having the lowest scores for trust in healthcare professionals, contact tracers, and governmental health authorities [42], as influenced by the significant socioeconomic adversities they faced during the COVID-19 pandemic. In addition, in a study among US Latinos and immigrants from Latin America, respondents who consumed Spanish-language news were more inclined to trust journalists as compared to those who consumed primarily English-language media [25]. Further, trust in Spanish-language journalists led to a better evaluation of state and local officials’ COVID-19 response and was associated with an increased likelihood of supporting collective health efforts [25].

### Correlates of Mental Health

A higher recurrence of anxiety and feelings of depression were reported among other participants compared to US Latino males who were above the poverty line [43]. Compared to individuals who were employed in a non-essential workplace setting, individuals who were unemployed reported a higher recurrence of anxiety and feelings of depression [24,25,26]. Further, individuals who were employed and were above the poverty line were more likely to live in single-family homes, have a higher education, and be married [43].

### Correlates of Self-Rated Health

Higher odds of poor or fair health among Latinos were associated with age, and higher levels of education and income were associated with lower odds of individuals’ reporting poor or fair health [44]. Even during pre-pandemic times, a significant association was found between increases in age and worse SRH among Mexican immigrants [14]. Further, lower levels of acculturation, as measured partly by the proportion of life lived in the US, were associated with worse SRH among Mexican immigrants [14]. Among Mexicans who reside in the US, foreign-born individuals demonstrated lower odds of reporting poor or fair SRH compared to individuals born in the US [45]. In addition, among Mexicans who reside in the US, married individuals had 14% lower odds of reporting poor or fair health relative to the unmarried [45].

### Summary

The objective of this study is to assess the associations between informational trust (formal and informal) and SRH and to explore the extent to which this relationship is mediated by mental health measures (anxiety, depression, and stress), controlling for demographic factors. In the context of the experience of Latino immigrants, exploring the association between mental health, SRH, and trust is particularly important, as Latino immigrants, including LDLs, have experienced a disproportionate impact of the COVID-19 pandemic. In addition, it is imperative to consider the relationship between trust and SRH, as the sources of information LDLs trust can inform their health decision-making behaviors, including whether to adopt and participate in healthier actions, such as the COVID-19 mitigation practices, which could then influence the perception of their health risk, ability, and confidence in managing their health and SRH outcomes. An understanding of the factors that influence SRH also can inform public health policies and interventions to decrease poor health outcomes and reduce health disparities among LDLs.

## Methods

### Background

A rapid needs assessment survey was conducted to characterize LDLs’ experience of the COVID-19 pandemic and to assess how stressors and protective factors experienced during the pandemic influenced the mental health of LDLs. The study was funded by the National Institute on Minority Health and Health Disparities and approved by the Committee for the Protection of Human Subjects of the University of Texas Health Science Center at Houston.

### Recruitment

Data were collected during November and December 2021. Participants were recruited in the Houston metropolitan area from day labor “corners” or locations where LDLs gather to seek work (i.e., parking lots of home improvement stores, convenience stores, gas stations, apartment complexes, public parks, and street intersections).

A corner sampling strategy was developed prior to recruitment. A list of 30 previously observed corners was stratified according to the number of LDLs observed. Corners with fewer than 15 LDL observed were classified as “small.” Corners at which 15 to 29 LDL were observed were classified as “medium.” Corners with 30 or more LDL were classified as “large.” Corners deemed to be adjacent (within approximately three blocks of each other) were considered as a single location and classified according to the aggregate number of LDLs observed across the individual corners. Of the 30 locations, 17 were small, 7 were medium, and 6 were large.

An overall goal of 300 participants was set for the study. Based on the prior observations, a goal of 80 participants each from small and medium locations and 140 from large locations was specified. Within each size strata, locations were randomly ordered using a random number generator. Corners within each stratum were visited in the randomly determined order. As many interviews as possible were conducted before moving to the next location.

Participants had to be 18 years of age or older, self-identify as Latino, be at the corner, seeking work, and have previously sought work at a corner. Trained interviewers explained the purpose of the study, determined LDL eligibility, and consented eligible candidates. Surveys were administered on iPads in Spanish. A total of 416 LDLs were observed, and 314 were approached at 18 corners. Of these, 304 consented, and 300 surveys were completed.

### Measures

*Demographicmeasures* included participant’s age, years in the US, income, years of schooling, country of birth, marital status, and the number of days in the last month that work was found at the corner. Those who had worked at least one day were asked how much they earned on a typical day. Thirty-day income was computed by multiplying days worked by typical daily earnings. Country of birth was coded as United States; Mexico; Central America (Honduras, Guatemala, El Salvador, or Nicaragua); Cuba; or other. Marital status was coded as single, never married; married or living with a partner; or formerly married (divorced, widowed, or separated). To reduce the skewness in the distribution of the income variable, seven values of monthly income greater than $3,000 (ranging from $3,150 to $9,600) were set to $3,000. For the analyses reported below, monthly income for each participant was divided by 100. Dummy variables were created for each category of marital status and country of birth, with “single” and “United States” used as the referral categories, respectively.

*Mental health* was measured by three previously validated scales. Depression was measured with the 7-item Center for Epidemiologic Studies Depression Scale (CES-D) [46]. A sample item is, “In the last week, how often would you say you didn’t feel like eating; your appetite was poor?” Responses were recorded on a 4-point scale, with 0 = not at all (less than 1 day); 1 = a little (1–2 days); 2 = frequently (3–4 days); 4 = a lot (5–7 days). Cronbach’s *α* for the scale was .87. *State anxiety* was measured by a six-item survey [47]. A sample item is, “In general today, do you feel nervous?” Three items that reflected a positive state (e.g., “calm”) were reverse scored. Responses were recorded on a four-point scale, with 0 = not at all; 1 = somewhat; 2 = moderately so; 3 = very much so. Cronbach’s α for the scale was .73.

*Stress* was measured using a six-item version of the Perceived Stress Scale [48]. A sample item is, “In the past 30 days, how often have you been upset because of something that happened unexpectedly?” Responses were recorded on a 5-point scale, with 0 = never; 1 = almost never; 2 = sometimes; 3 = fairly often; 4 = very often. Cronbach’s *α* for the scale was .88. For each scale, the scale score was computed as the mean value of non-missing responses.

*Trust* in sources of information was measured by 15 items, developed for this study. The scale was informed by a study conducted by the University of Chicago National Opinion Research Center. Using the item, “How much do you trust the Coronavirus (COVID-19) information disseminated by the following sources of information?” sources included television networks, conversations with family or friends or coworkers, and social media. Responses were recorded using a 4-point scale, with 0 = no trust. 1 = some trust; 2 = a great deal of trust; 3 = a lot of trust. Participants could refuse to answer any item or indicate that they did not know how much they trusted a given source or that a particular source was not applicable for them.

An exploratory factor analysis of the trust items was conducted using principal axis factoring extraction and oblimin rotation. Three factors (“Spanish and other networks”, “other media”, and “other sources”) were extracted, which accounted for 47.2%, 7.3%, and 7.0%, respectively, of the variance in the items. From the first factor, four items were selected that reflected Spanish-language television networks: Univision, Telemundo, CNN/CNN in Spanish, and Fox News/Fox News in Spanish (“Spanish and other networks”). Five items from the second factor reflected other media sources and sources of information: radio stations, WhatsApp groups, official press releases or conferences, daily or weekly newspapers, and social media (“other media”). Four items were selected from the third factor: conversations with friends or coworkers, opinion polls, conversations with family, and websites or online news sites (“other sources”).

After the research team reviewed these three factors, two trust scales were formulated. Trust in formal sources of information (*formal trust*) consisted of trust in Telemundo, Univision, CNN, Fox, newspapers, and radio stations. Trust in informal sources of information (*informal trust*) consisted of conversations with friends or coworkers, conversations with family, websites or online news, social media, and WhatsApp. Trust in Telemundo and Univision was originally assessed in a single item. The field team, however, reported that participants made a distinction between the two sources, and separate items for each source were created. These items were used for analyses. Thus, there were fewer responses to these items in comparison to the trust items. Excluded trust variables included trust in PBS, official press releases or conferences, medical professionals, and opinion polls. For the analyses presented below, response options of “a great deal” and “a lot” for the trust items were combined. Responses of “refused,” “don’t know,” and “not applicable” were treated as missing responses. Again, scores were computed as the mean response of non-missing items. Cronbach’s *α* for the formal trust items, using the recoded items, was .88; Cronbach’s *α* for the informal trust items was .81.

Notably, a missing value for at least one scale item resulted in the exclusion of 127 cases for computation of *α* for formal trust and the exclusion of 57 cases for informal trust. The mean anxiety score was higher for those cases used to compute formal trust *α* than those not used (.84 vs. .66; independent-samples t = −2.55; *df* = 269; *p* =.011). The mean age for cases used to compute informal trust *α* was 44.1 compared to 50.0 for cases not used (*t* = 3.31; *df* = 269; *p* < .001). Mean years of school for cases used to compute informal trust *α* was higher than for cases not used (8.1 vs 6.1; *t* = −3.08; *df* = 269; *p* = .001) and similarly for mean SRH (1.98 vs 1.56; *t* = −2.23; *p* = .013). One-half (51.9%) of cases used to compute alpha for informal trust included participants who were single, never married as compared to 29.8% of cases not used (Pearson χ^2^ = 8.78; *df* = 1; *p* =.003). One-third (33.6%) of cases used to compute *α* for informal trust included participants who were married or living with a partner as compared to 52.6% of cases not used (*χ*^2^ = 6.91; *df* = 1; p = .009).

*Self-rated health* was measured by a single item, “In general, would you say your health is . . . ?” Responses were measured on a 5-point scale with 0 = poor; 1 = fair; 2 = good; 3 = very good; 4 = excellent [11,14]. Scale scores were computed as the mean score of non-missing responses across the items in the scale.

### Data Analysis

Descriptive statistics were computed for the study measures using SPSS version 29.0. Means and standard deviations were computed for continuous variables, such as age and number of days worked. Frequencies were computed for marital status.

**Figure 1:**
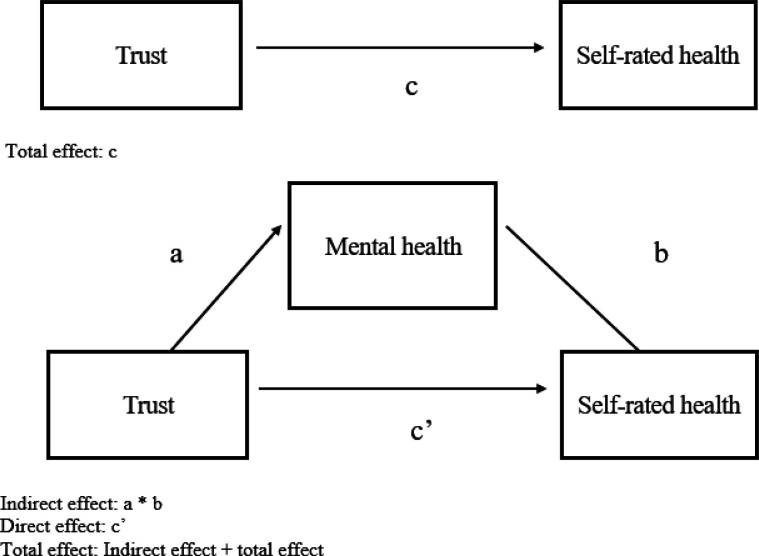
SRH Model

As noted, we were interested in assessing the association between each informational trust measure and SRH and whether any associations were mediated by the individual mental health measures. Our SRH model of interest is presented in Figure 1. To assess the model, we applied the product of coefficients approach [49]. Using Hayes’ PROCESS version 4.2 in SPSS, we generated linear regression models simultaneously to assess the total effect of trust (X) on SRH (Y), shown by path c; the effect of trust on the mental health mediator (M), shown by path a; and the effect of the mediator on SRH, controlling for trust, shown by path b. The demographic characteristics were entered as covariates.

The total effect was partitioned into an indirect effect given by the product of a and b and a direct effect, represented by c’. With rounding, the total effect will equal the sum of the indirect and direct effects. Mediation is present if a significant indirect effect is observed. Given two measures of trust (formal and informal) and three measures of mental health (depression, anxiety, and stress), six separate analyses were run for each possible combination of trust and mental health. Although the results are presented in terms of “effects,” the data considered here are from a correctional study and are not to be considered causal. A significance level of *p* < .05, two-tailed was used for analyses.

## Results

The overall sampling goal was met, with a total of 300 LDLs surveyed. Strata-specific goals also were met, with 80 participants recruited from six small corners, 80 recruited from six medium corners, and 140 recruited from six large corners. A total of 271 of 300 (90.3%) participants had complete data for each of the study variables and formed the sample for this study. Characteristics of the sample are shown in Tables 1 and 2. On average, participants were in their mid-40s, had been in the US for approximately 14 years, and had completed approximately eight years of schooling. On average, participants had earned approximately $900 in the 30 days prior to their survey. Nearly one-half had never been married, and nearly two-fifths were married or living with a partner.

Mean values of the trust measures indicated an average response of “some trust” in the items. The mean score for depression indicated an average frequency of “a little,” based on the response options reported above. The mean score for anxiety indicated an average response of “somewhat” to the items. The mean stress score indicated an average response between “almost never” and “sometimes.” The mean SRH score indicated an average level of SRH of “good.” There were no significant differences in these characteristics between those with complete data and those without.

Frequencies of the individual formal and informal trust items are shown in Table 3. For each item, the frequency of each possible response is presented in the first row of the table. The second row presents percentages for each valid response, i.e., excluding responses of “refused,” “don’t know,” and “not applicable.” The third row presents percentages for all possible responses.

Table 4 presents the results of the mediation analyses with unstandardized regression coefficients and 95% confidence intervals. As shown in the table, for each of the models of formal trust and SRH (Models 1 through 3), there was a significant and inverse total effect of formal trust on SRH (corresponding to path c in Fig. 1). On average, the greater one’s trust in formal sources of information, the lower one’s SRH. In considering depression as a potential mediator of this association (Model 1), there was a significant and inverse association between depression and SRH (path b). A greater level of depression was associated with lower SRH. There was no significant association, however, between formal trust and depression (path a). Consequently, there was no significant indirect effect of formal trust on SRH through an association with depression (a * b). A significant and inverse direct effect of formal trust on SRH (path c’) remained after adjusting for the indirect effect.

A similar result was found when considering formal trust and anxiety (Model 2). Although there was an association between increased anxiety and lower SRH, there was no association between formal trust and anxiety, and no significant indirect effect was observed. A significant direct effect was observed. In Model 3, formal trust was not significantly associated with stress. Greater stress was associated with lower SRH, but this association was not significant at the *p* < .05 level, and there was no significant indirect effect. There was a significant direct effect of formal trust on SRH.

As shown in Models 4 through 6 in Table 4, there were no significant total or direct effects between informal trust on SRH. Depression and anxiety were inversely associated with SRH. Greater stress was associated with lower SRH, but this association was not significant at the *p* < .05 level. Informal trust was not associated with any of the measures of mental health, and there were no significant indirect effects.

## Discussion

This study assessed the association between trust in sources of COVID-19 information (formal and informal) and SRH and to explore the extent to which this relationship was mediated by mental health, controlling for demographic factors. In this study, we found two independent pathways to SRH: trust and SRH and mental health and SRH. We found that greater trust in formal sources of information was associated with decreased SRH. Greater levels of anxiety and depression also were associated with decreased SRH. There were no significant associations between formal trust and mental health measures. Thus, the association between formal trust and decreased SRH was not mediated by mental health. In addition, trust in informal sources of information was not significantly associated with SRH or mental health. Higher levels of depression and anxiety, however, were associated with lower SRH.

Our results suggest that the information received during the COVID-19 pandemic led to lower SRH due to the negative content of the information about COVID-19 that was portrayed in broadcast media channels, including Univision and Telemundo. Although the generalized negative tone of the COVID-19 information was not exclusive to these media sources, the reasons that lower SRH was related to information exposure through media channels that are not trusted need to be explored further. Previous evidence indicates that individuals who seek general health information tend to report better SRH, while individuals who seek disease-specific information report worse SRH [50]. This pattern of findings also may contribute to the larger discussion of the ways that the nature, tone, and quality of information have the capacity to influence not only knowledge and awareness of health problems but also the perceived well-being of individuals.

Our study has several strengths, including our rigorous sampling and recruitment process, as well as our timely data collection, which was captured at the time that COVID-19 variants and subvariants were of utmost concern and precautions were still encouraged. Because this is a cross-sectional study, however, it was carried out at a specific point in time and cannot lead to causal inferences [51].

## Conclusions

The results from our current study contribute to the literature by demonstrating that formal trust and mental health appear to be associated with SRH among LDLs. Individuals’ trust in formal and informal sources merits further exploration, as such sources can provide different types of information that lead to health behaviors and personal assessments that may ultimately influence perceived well-being. Although our focus was on the trust of information sources, future research also should examine how the frequency of news exposure affects the trust in information and its effect on SRH. Our findings on formal sources of information, however, also may be applicable to other vulnerable populations with low levels of trust in the media channels to which they are exposed, and the impact on their well-being may extend to issues beyond the COVID-19 pandemic.

## Figures and Tables

**Table 1:** Country of Birth and Marital Status (*N* = 271)

Variable	*n*	%
Country of birth		
United States	8	3.0%
Central America	122	45.0%
Mexico	101	37.3%
Cuba	33	12.2%
Other	7	2.6%
Marital status		
Single, never married	128	47.2%
Married, living with a partner	102	37.6%
Formerly married	41	15.1%

**Table 2:** Other Demographic Characteristics (*N* = 271)

Variable	Possible range	Observed range	*M*	*SD*
Age		18.4–76.1	45.3	12.1
Years in the US		.08–59.3	13.7	12.2
Years of school		0–22.0	7.7	4.4
30-day income		0–2,500.00	906.79	691.10
Trust				
Formal	0–2.0	0–2.0	1.2	0.6
Informal	0–2.0	0–2.0	1.1	0.5
Mental health				
Depression	0–3.0	0–3.0	0.9	0.7
Anxiety	0–3.0	0–2.7	0.8	0.6
Stress	0–4.0	0–4.0	1.6	0.9
Self-rated health	0–4.0	0–4.0	1.9	1.3

**Table 3:** Trust in Sources of Information Items

Trust variable	No	Some	A great deal/a lot of	Total valid cases	Not applicable	Donť know	Refused	Total

CNN/CNN in Spanish	33	100	70	203	52	15	1	271
Valid %	16.3%	49.3%	34.5%	100.1%	-	-	-	-
Total %	12.2%	36.9%	25.8%	-	19.2%	5.5%	0.4%	100.0%

Fox News/Fox News in Spanish	42	85	65	192	61	18	0	271
Valid %	21.9%	44.3%	33.9%	100.1%	-	-	-	-
Total %	15.5%	31.4%	24.0%	-	22.5%	6.6%	0.0%	100.0%

Telemundo	34	94	86	214	12	4	2	232
Valid %	15.9%	43.9%	40.2%	100.0%	-	-	-	-
Total %	14.7%	40.5%	37.1%	-	5.2%	1.7%	0.9%	100.1%

Univision	26	95	100	221	10	1	0	232
Valid %	11.8%	43.0%	45.2%	100.0%	-	-	-	-
Total %	11.2%	40.9%	43.1%	-	4.3%	0.4%	0.0%	99.9%

Daily or weekly newspapers	56	103	58	217	41	13	0	271
Valid %	25.8%	47.5%	26.7%	100.0%	-	-	-	-
Total %	20.7%	38.0%	21.4%	-	15.1%	4.8%	0.0%	100.0%

Radio stations	53	120	73	246	22	3	0	271
Valid %	21.5%	48.8%	29.7%	100.0%	-	-	-	-
Total %	19.6%	44.3%	26.9%	-	8.1%	1.1%	0.0%	100.0%

Conversations with family	33	97	138	268	2	1	0	271
Valid %	12.3%	36.2%	51.5%	100.0%	-	-	-	-
Total %	12.2%	35.8%	50.9%	-	0.7%	0.4%	0.0%	100.0%

Conversations with friends or coworkers	59	116	95	270	1	0	0	271
Valid %	21.9%	43.0%	35.2%	100.1%	-	-	-	-
Total %	21.8%	42.8%	35.1%	-	0.4%	0.0%	0.0%	100.1%

Websites or online news sites	56	109	61	226	34	10	1	271
Valid %	24.8%	48.2%	27.0%	100.0%	-	-	-	-
Total %	20.7%	40.2%	22.5%	-	12.5%	3.7%	0.4%	100.0%

Social media	81	105	57	243	21	7	0	271
Valid %	33.3%	43.2%	23.5%	100.0%	-	-	-	-
Total %	29.9%	38.7%	21.0%	-	7.7%	2.6%	0.0%	99.9%

WhatsApp groups	102	100	37	239	27	5	0	271
Valid %	42.7%	41.8%	15.5%	100.0%	-	-	-	-
Total %	37.6%	36.9%	13.7%	-	10.0%	1.8%	0.0%	100.0%

**Table 4:** Mediation Analysis

Model	Path (Fig. 1)	*B*	95% confidence interval
Model 1			
Formal trust → depression	a	.106	(−.042, .255)
Depression → self-rated health	b	− .383	(−.591, −.176)
Formal trust → self-rated health	c’	− .287	(−.541, −.032)
Indirect effect	a * b	− .041	(−.113, .025)
Direct effect	c’	− .287	(−.541, −.032)
Total effect	c (indirect + direct)	− .327	(−.587, − .068)
Model 2			
Formal trust → anxiety	a	− .046	(−.167, .074)
Anxiety → self-rated health	b	− .487	(−.744, −.231)
Formal trust → self-rated health	c’	− .350	(−.604, − .096)
Indirect effect	a * b	.023	(−.035, .093)
Direct effect	c’	− .350	(−.604, − .096)
Total effect	c (indirect + direct)	− .327	(−.587, − .068)
Model 3			
Formal trust → stress	a	.040	(−.152, .233)
Stress → self-rated health	b	− .156	(−.319, .007)
Formal trust → self-rated health	c’	− .321	(−.580, − .063)
Indirect effect	a * b	− .006	(−.051, .029)
Direct effect	c’	− .321	(−.580, − .063)
Total effect	c (indirect + direct)	− .327	(−.587, − .068)
Model 4			
Informal trust → depression	a	.068	(−.091, .227)
Depression → self-rated health	b	− .405	(−.641, −.196)
Informal trust → self-rated health	c’	.031	(−.242, .305)
Indirect effect	a * b	− .028	(−.104, .041)
Direct effect	c’	.031	(−.242, .305)
Total effect	c (indirect + direct)	.004	(−.277, .284)
Model 5			
Informal trust → anxiety	a	− .025	(−.153, .104)
Anxiety → self-rated health	b	− .471	(−.730, −.212)
Informal trust → self-rated health	c’	− .008	(−.282, .267)
Indirect effect	a * b	.012	(−.052, .083)
Direct effect	c’	− .008	(−.282, .267)
Total effect	c (indirect + direct)	.004	(−.277, .284)
Model 6			
Informal trust → stress	a	− .026	(−.232, .180)
Stress → self-rated health	b	− .161	(−.326, .003)
Informal trust → self-rated health	c’	.000	(−.280, .279)
Indirect effect	a * b	.004	(−.036, .046)
Direct effect	c’	.000	(−.280. .279)
Total effect	c (indirect + direct)	.004	(−.277, .284)

*Note. B=* Unstandardized regression coefficient.

## Data Availability

The datasets used and/or analyzed during the current study are available from the corresponding author upon reasonable request.

## Abbreviations

SRH: Self-Rated Health
LDLs: Latino Day Laborers
COVID-19: Coronavirus Disease 2019
CNN: Cable News Network
CES-D: Center for Epidemiologic Studies Depression Scale
